# Artificial-Weld-Crack Detection Network, YOLOv6-NW, Based on Target Recognition Technology

**DOI:** 10.3390/ma17246102

**Published:** 2024-12-13

**Authors:** Yiming Wang, Lunhua Shang, Bin Li, Yu Liu, Ye Ji, Linbo Hao, Yang Zhang, Yuchen Li, Menghan Tian

**Affiliations:** 1 Luoyang Institute of Science and Technology, Luoyang 471023, China; 200900201830@lit.edu.cn (Y.W.); libin@lit.edu.cn (B.L.); 218104010147@njust.edu.cn (Y.L.); kerwin@lylgxyznzzxy.wecom.work (Y.J.); lb_hao@nuaa.edu.cn (L.H.); 200900401815@lit.edu.cn (Y.Z.); 18239545548@163.com (Y.L.); tian11071885@163.com (M.T.); 2 School of Physics and Electronic Science, Guizhou Normal University, Guiyang 550001, China

**Keywords:** target recognition, weld crack, YOLOv6, defect detection, nondestructive testing

## Abstract

Aiming at the problems of scarce datasets and the low identification accuracy faced in the field of weld-crack detection, this paper proposes an artificial-weld-crack preparation method based on the doping of dissimilar metal particles to augment the number of samples of weld-crack defects. Meanwhile, data augmentation methods such as random cropping, scaling and Mosaic are combined to further enhance the richness of the samples, so as to provide strong data support for the proposed weld-crack-defect detection model. Given the limitations of storage and computational resources in industrial application scenarios, this paper designs the lightweight detection network YOLOv6-NW. It is achieved by optimizing the YOLOv6-N model for width and depth compression to efficiently identify and locate weld-crack defects. The experimental results demonstrate that YOLOv6-NW significantly outperforms YOLOv5 in terms of both model detection performance and model size. Compared to YOLOv6-N, the number of model parameters in YOLOv6-NW is only 16% of that in YOLOv6-N, yet its model detection accuracy and recall rate remain on a comparable level with YOLOv6-N. Additionally, the precision of crack detection by YOLOv6-NW under low-resolution or low-illumination conditions remains above 0.9.

## 1. Introduction

The welding process, due to its multi-parametric, strong coupling, nonlinear and time-varying characteristics, coupled with complex environmental factors such as the strong arc light, ultra-high temperature, electromagnetic interference, dust and splash, brings great difficulties to the real-time monitoring and evaluation of welding behavior [1]. However, in large-scale production, in order to ensure the quality of welding, and evaluate and optimize the welding process, real-time and accurate monitoring and analysis of the information in the welding process is particularly important. Therefore, in-depth research on how to effectively resist external interference in the welding process and achieve efficient and accurate monitoring of the welding process is of extremely important practical significance and value [2].

The reasonable setting of process parameters has a decisive impact on the quality of the deposited layer during the welding process. For example, improper settings of parameters such as the welding current, voltage, path trajectory, welding speed and wire feeding speed may significantly affect the quality of the deposited layer. The defects of the deposited layer mainly include two types: quality defects and forming defects. Quality defects include porosity, cracks, non-fusion, slag inclusion, welding nodules, etc. Forming defects include serious thermal deformation, collapse, and geometrical size deviation of the weld width and reinforcement [3]. Defects in welding cause serious damage to the overall performance of the weld, which may hinder the perfect forming of the weld, weaken its connection strength and lead to uneven stress distribution [4]. What is more serious is that these defects may become a safety hazard in the actual application of metal workpieces, which may lead to unexpected serious accidents.

At present, defect detection technology can be broadly divided into two categories. One is the traditional physics-based nondestructive detection technology, such as magnetic-particle detection, ultrasonic detection, eddy current detection, penetration detection and X-ray detection. The other is the defect detection technology based on machine learning (including deep learning) [5].

The traditional physics-based nondestructive testing technology has a clear realization principle, and the test results are more interpretable, but there are some drawbacks. For example, Chen et al. proposed a dry-magnetic-particle detection method based on thermal imaging, which identifies cracks by the heat distribution during the formation of magnetic marks [6]. Compared with traditional magnetic-particle detection, it has a wider detection range, and not only retains the excellent detection ability of the traditional magnetic-particle detection on micro-gap cracks but can also detect large-scale cracks more effectively, but the cost is higher. Liu et al. developed a dual-laser ultrasonic system for crack detection by comparing the spectral responses of the material under test between the broadband input and dual inputs of both broadband and narrowband signals. This system achieves high accuracy but involves complex operations [7]. Malarvel et al. proposed a novel anisotropic diffusion model to reduce the noise of X-ray images while preserving the detailed information [8].

With the rapid progress of machine learning technology, a variety of traditional machine learning algorithms such as Support Vector Machine (SVM), Random Forest (RF), etc., as well as deep neural networks have been widely used in the field of weld-defect detection. Hu et al. proposed an innovative convolutional neural network model based on an object-level attention mechanism to accurately detect tiny casting defects in radiographic images [9]. In addition, they further enhanced the interpretability of the model by introducing bilinear class activation map technology, making the detection results clearer. Munir et al. used autoencoder deep neural networks to process the original ultrasonic signals and effectively filter out the noisy signals therein, thus improving the accuracy of deep learning classifiers in the task of welding-defect classification [10]. Wu et al. designed a fluorescent magnetic-particle detection scheme based on the Scale-YOLOv4 model, which improved the automatic detection efficiency of weld cracks and realized accurate positioning in three-dimensional space. The average precision of crack detection is 85.4%, and the average absolute error is less than 1 mm [11].

With the vigorous development of a new generation of artificial intelligence technology, the field of intelligent welding-defect detection has ushered in unprecedented opportunities [12]. Compared with traditional physics-based nondestructive testing technology, the defect detection method based on machine learning shows more-significant advantages of integrating into an intelligent welding manufacturing system with its characteristics of low cost and a high automation level [13]. Under the background of “Made in China 2025” and “Industry 4.0” intelligent manufacturing, this paper focuses on the crack detection technology based on target recognition in general welding operations, aiming to solve the key problems of quality detection in intelligent manufacturing. This paper explores a new method of crack-defect detection in the welding process, which provides strong technical support and reliable quality assurance for large-scale, efficient and intelligent welding operations.

## 2. Weld-Crack-Defect Data Preparation

### 2.1. Preparation Method for Non-Natural Weld-Seam Cracks

A weld crack is one of the most common defects in the arc-welding process, which not only affects the appearance and forming quality of the weld, but also weakens the joint strength of the weld [14]. In the process of arc additive manufacturing, if the weld cracks on the surface of the historical deposit layer are not found and treated in time, these cracks may develop into internal weld cracks, which has a serious impact on the overall quality and service life of the metal component.

In view of the difficulty in obtaining a large number of weld-crack-defect samples in the real working environment, people began to adopt the method of artificially manufacturing welding defects to obtain the samples needed for research. For example, the high-tech Jining Dongfang Mould Manufacturing Co., Ltd. has obtained a utility model patent for preparing artificial cracks in welding-defect test blocks [15]. Wuhan University also published a patent for an invention that creates defects on the surface of test blocks by mechanical indentation or chemical etching, and connects different test blocks by vacuum diffusion welding to produce additive manufacturing workpieces with built-in artificial defects [16]. This paper also uses the method of manual intervention in the arc-welding process to simulate the generation of crack defects. This manual intervention includes the deliberate introduction of dissimilar particles in the welding process, the deliberate adjustment of welding parameters to an unfavorable state, and the change in the distance between the conductive nozzle and the workpiece. In this way, the possible crack defects in actual welding are simulated. 

**Figure 1:**
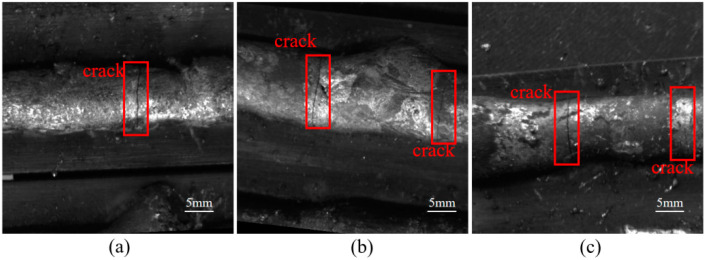
Weld-seam cracks caused by doping with silicon particles. (**a**) Example One; (**b**) Example Two; (**c**) Example Three.

Figure 1 shows the unnatural weld cracks induced by the intentional doping of dissimilar particles, such as silicon and manganese, during arc welding. In order to carry out this experiment, the following basic conditions were set: DC welding mode, the welding current set to 300 A and the welding speed set to 6 mm/s. The welding wire model was ER316L, with a diameter of 1.2 mm and a substrate of 304 stainless steel. In this process, silicon (more than 99.40% purity) and manganese (more than 99.98% purity) were used as experimentally optional doped dissimilar particles. For single-pass, single-layer welding or multi-pass, single-layer welding, different particles can be added in a random and dispersed manner on the pre-set welding path. For single-pass, multi-layer welding or multi-pass, multi-layer welding, a follow-up powder-feeding device can be used to mix the required heterogeneous particles dynamically and in real time during the arc-welding process. The experimental results indicate that although doping with manganese particles can increase the occurrence of unnatural weld cracks to a certain extent, it will significantly affect the gloss of the weld surface, resulting in a significant visual difference between unnatural cracks and natural cracks. However, when the doped dissimilar particles were changed from manganese to silicon, not only was the yield of unnatural weld cracks improved, but the shape and gloss of the weld surface were also less affected by silicon particles. Therefore, in this experiment, silicon was ultimately chosen as the heterogenous particle for doping in arc welding.

Considering the complexity and variability of a real welding operation environment, special attention should be paid to the diversity and richness of datasets when using deep learning to train neural network models. In order to enhance the robustness of the weld-crack-defect detection model under various operating conditions, a Basler 1920-155 µm camera with a resolution of 1920 × 1200 pixels was used in the experiment. The images of weld cracks were captured under different illumination conditions and shooting angles to simulate the complex actual arc-welding environment. Specifically, the arc-welding experiment produced a total of 60 metal workpieces with cracks, and 500 raw weld-crack images were collected by an industrial camera.

### 2.2. Data Annotation and Data Augmentation

When training a supervised-learning-based weld-crack-defect detection model, accurate labels of the samples are required, which involves determining the exact bounding box coordinates of the weld crack in the image. For this purpose, this paper uses the LabelImg tool to label the weld-crack data. LabelImg-v1.8.0 is a lightweight and easy-to-use image annotation software that can save annotation data in a VOC_XML or YOLO-TXT format. In the experiment, the VOC_XML format was selected to output the weld-crack boundary box information marked by the LabelImg tool.

On the production line, due to the low probability of the occurrence of defects in workpieces, it becomes a challenge to collect a sufficient number of defect samples to train a supervised learning model. In contrast, normal (non-defective) samples are relatively easy to obtain. As a result, researchers have turned to unsupervised learning methods that attempt to train defect detection models using only normal samples. Unsupervised defect detection usually relies on frameworks such as self-encoders or generative adversarial networks. However, defect detection sometimes shows large differences between normal samples, but small differences between normal samples and defective samples. The performance of the unsupervised learning model based on reconfiguration error is not ideal.

Direct training of a supervised learning model with a limited number of small samples can easily lead to overfitting, which weakens the generalizability of the model. To cope with this challenge, the current mainstream methods for solving the sample sparsity problem include data augmentation or data generation, pre-training and migration learning, and rational design of the network structure [17].

**Figure 2:**
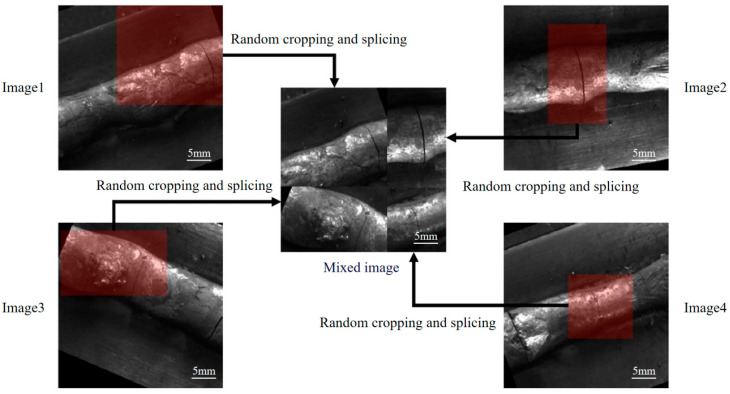
Example of Mosaic data augmentation.

In order to improve the accuracy of the weld-crack-defect detection model on limited datasets, this paper adopts data augmentation techniques to effectively expand the number of defect samples, thereby enriching the diversity of the training set. There are various methods for data augmentation. These include geometric transformations like horizontal or vertical flipping, rotation, cropping, and translation. Color-gamut or color-adjustment techniques are also used, such as color jitter, edge enhancement and changes in lighting effects. Other methods involve adding noise interference and random pixel erasure. In addition, mixing images, generating adversarial networks and style transfer are also methods of data enhancement. In order to improve the robustness of the model to the dataset based on the characteristics of weld-crack-defect images, this paper adopts various data augmentation strategies to expand the original dataset. In addition, Mosaic data augmentation technology has been introduced to create more-diverse training samples by combining multiple images [18]. As is shown in Figure 2, the Mosaic data augmentation algorithm creates a new mixed image by randomly cropping and concatenating four images. This data augmentation strategy not only provides a richer background environment for object detection tasks, but also helps the model identify weld-crack defects in complex backgrounds. Moreover, by processing multiple images at once, the efficiency of batch normalization operations is significantly improved, thereby accelerating the training process of the model.

## 3. Weld-Crack-Defect Detection Network

### 3.1. Structure of the Weld-Crack-Defect Detection Model

In industrial applications, model deployment environments require object detection algorithms to achieve a good balance between detection accuracy and speed. To meet this demand, YOLOv6, as a single-stage object detection framework designed specifically for industrial scenarios, has demonstrated its outstanding performance. This framework supports deployment on various hardware platforms such as GPUs, CPUs and ARM to cope with diverse industrial environments. In order to adapt to the limitations of different hardware resource conditions, YOLOv6 provides a variety of model sizes. It includes the nano model YOLOv6-N, the small model YOLOv6-S, the medium model YOLOv6-M and the large model YOLOv6-L. These models of different sizes can be flexibly selected according to the hardware conditions and performance requirements of specific application scenarios, thus ensuring an efficient detection speed while ensuring detection accuracy. According to the characteristics of the application scenarios of weld-crack-defect detection and the actual detection requirements, this paper proposes a weld-crack-defect detection model, YOLOv6-NW, based on the improved YOLOv6-N. This improved model achieves a more lightweight design while maintaining a high performance, allowing the model to be successfully deployed and applied in a wider range of industrial environments.

**Figure 3:**
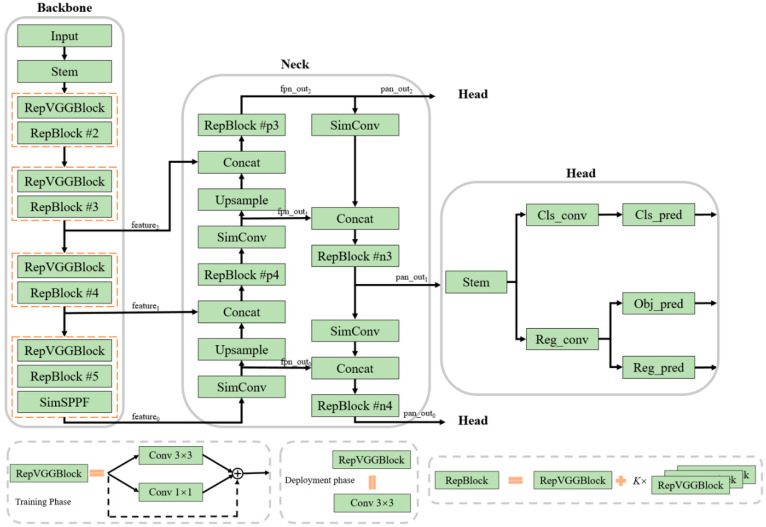
YOLOv6-NW model structure.

In order to develop lighter-weight industrial-application-level models, this paper compresses the model size by reducing the model width and model depth. Specifically, in terms of model width, the strategy of halving the number of channels is adopted, so that the number of channels in each layer of the neural network of the proposed YOLOv6-NW model is significantly reduced compared with that of YOLOv6-N. In terms of model depth, the RepBlock structure in YOLOv6-N is optimized to appropriately reduce the number of repetitive units RepVGGBlock. These specific differences are shown in Table 1. As is shown in Figure 3, RepBlock consists of a basic RepVGGBlock and several stacked RepVGGBlocks, where the RepVGGBlock structure draws on the idea of structure reparameterization in the convolutional neural network RepVGG. Structural reparameterization is a strategy to enhance feature extraction capabilities in model training, which utilizes a multi-branch architecture to provide a rich model representation during the training phase. And, in the model inference stage, the original multi-branch model is transformed into a more-efficient single-path model through parameter equivalence transformation. Thus, a small additional model training cost can be used in exchange for stronger model performance under the same inference time. In this paper, by moderately removing some of the similar cell structures, the complexity of the model can be significantly reduced without compromising the performance of the model. This strategy is the core reason for deciding to streamline the number of neural network layers on the RepBlock unit structure.

As is shown in Figure 3, the structure of YOLOv6-NW, the weld-crack-defect detection model based on target recognition proposed in this paper, is mainly composed of three key parts: Backbone, Neck and Head. Among them, Backbone assumes the important role of feature extraction, while Neck is responsible for the effective fusion of low-level features with high-level semantic features by constructing a feature pyramid. The Head module is responsible for predicting the category, specific location and confidence level of the target object. Upon comparing the mainstream Backbone networks, it is found that RepVGG demonstrates superior feature representation on lightweight models while maintaining similar inference speeds. Therefore, in this paper, RepVGGBlock is used to construct the Backbone basic structure RepBlock. This paper adopts SimSPPF, a variant of Spatial Pyramid Pooling (SPP). SimSPPF replaces the SiLU activation function in SPPF with the ReLU activation function, which achieves faster model inference and further enhances the utility and efficiency of the model. Similarly, structural reparameterization is also used in Neck. On the basis of the improved PAN structure proposed in YOLOv5, the original CSPBlock is replaced by RepBlock to improve the model performance. Similar to YOLOX, Head adopts a decoupled structure and is based on a hybrid channel strategy to further minimize the extra latency overhead caused by convolutional operations while maintaining the accuracy of the model.

### 3.2. Loss Function and Model Optimization

**Figure 4:**
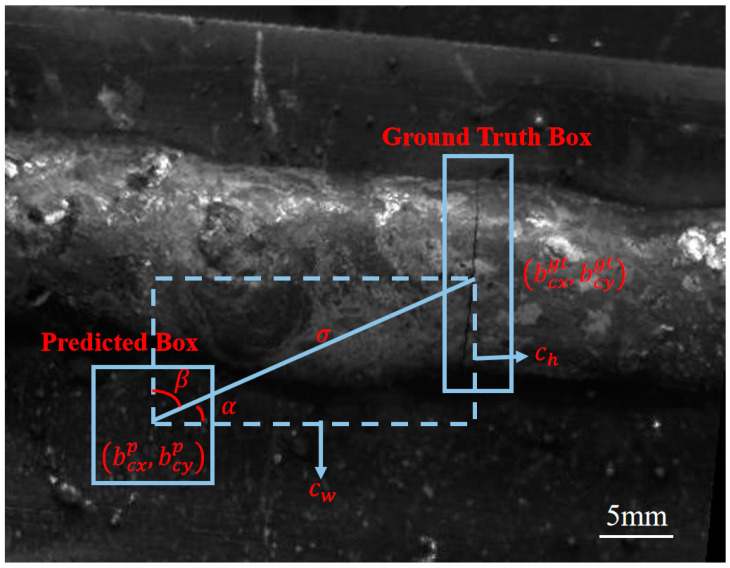
Schematic diagram of angle perception penalty terms.

The mainstream Anchor-free paradigm, SimOTA label assignment strategy and SIoU bounding box regression loss are used in the model optimization strategy. Compared to OTA, SimOTA obtains the best sample matching based on the Top-K approximation strategy, greatly reducing model training time. The traditional object detection loss function usually only selects one or more of the following: center distance, overlapping area and aspect ratio of the predicted and true boxes, as the model penalty indicators, while ignoring the importance of the angle on the bounding box regression. In this paper, SIoU is chosen as the bounding box regression loss function, which not only takes into account the common factors of the center distance, overlap region and aspect ratio, but also introduces an angle-aware penalty term in particular [19]. It can quickly move the predicted box to the nearest axis (*X*-axis or *Y*-axis) to the ground truth box during the optimization process. Subsequently, it continuously approaches the ground truth box along this axis, thereby improving the accuracy and efficiency of the bounding box regression. The schematic diagram of the angle perception penalty terms is shown in Figure 4. When α≤π/4, the convergence process tends to minimize α. Otherwise, the convergence process will tend to minimize β=π/2−α. The calculation method of the angle perception penalty term Λ is shown in Equations (1)–(4).

(1) $$\Lambda =1-2\sin^{2}\left(\arcsin x-\frac{\pi }{4}\right)$$

where x is determined by Equations (2)–(4).

(2) $$x=\frac{c_{h}}{\sigma }=\sin\alpha$$

(3) $$\sigma =\sqrt{{\left(b_{cx}^{gt}-b_{cx}^{p}\right)}^{2}+{\left(b_{cy}^{gt}-b_{cy}^{p}\right)}^{2}}$$

(4) $$c_{h}=\max\left(b_{cy}^{gt},b_{cy}^{p}\right)-\min\left(b_{cy}^{gt},b_{cy}^{p}\right)$$

where $b_{cx}^{gt}$ and $b_{cy}^{gt}$ represent the coordinates of the true box center point, while $b_{cx}^{p}$ and $b_{cy}^{p}$ represent the coordinates of the predicted box center point.

The complete expression of the SIoU loss function is shown in Equation (5), where the angle perception penalty term Λ is implicit in the expression of the distance penalty term Ω.

(5) $$L_{\mathrm{SIoU}}=1-IoU+\frac{\Delta +\Omega }{2}$$

## 4. Experimental Results and Analysis

Unlike research-oriented deep-neural-network model development, neural network models for industrial implementation must take into account, for example, the arithmetic level and storage capacity of the deployed platforms as well as the impact of various complex and variable external disturbances in real industrial scenarios. Model inference is often accelerated by reducing the resolution of the input image. While this is effective in reducing inference time, it comes with the risk of a reduced model performance. Therefore, balancing the relationship between input image resolution and model performance becomes a crucial task in industrial neural network modeling applications. In addition, the actual performance of the model in industrial application scenarios is also closely related to the image quality. However, actual industrial operating environments are often complex and harsh, such as in low-light environments where images captured by industrial vision sensing systems are of low illumination, low contrast and low quality. Therefore, industrial-application-grade models must ensure a stable and reliable performance under different operating conditions (e.g., image resolution, image quality, etc.).

In order to accurately evaluate the actual performance of the proposed YOLOv6-NW weld-crack-defect detection model in real welding environments, tests are performed on input images with different resolutions and different illuminations, respectively. The input image resolution includes 256 × 256 pixels, 512 × 512 pixels, 640 × 640 pixels and 768 × 768 pixels, and the set input image illumination is divided into three levels: low, medium and high. The three levels of low, medium and high are obtained by adjusting the exposure time. The exposure time for low-illumination images is 50 ms, for medium-illumination images it is 150 ms, and for high-illumination images it is 300 ms.

Three key evaluation metrics are chosen for this experiment, which are precision (P), recall (R) and mAP@0.5 [20]. The specific calculation formula is as follows:

(6) $$\mathrm{Precision}=\frac{TP}{TP+FP}$$

(7) $$\mathrm{Recall}=\frac{TP}{TP+FN}$$

(8) $$mAP=\frac{{\sum_{i=1}^{N}AP}_{i}}{M}$$

Here, *TP* stands for True Positives, which means correctly predicting positive instances as positive. *FP* stands for False Positives, which means incorrectly predicting negative instances as positive. *FN* stands for False Negatives, which means incorrectly predicting positive instances as negative. *N* represents the number of images tested. *M* represents the total number of categories used for detection. *AP* is the area under the PR curve. The mAP@0.5 (mean average precision, IoU = 0.5) is calculated by computing the *AP* for all images of each category when the IoU (Intersection over Union) threshold is set to 0.5, and then averaging these *AP* values across all categories. The IoU is the ratio of the intersection to the union of the predicted bounding box and the ground truth bounding box.

To make the statistical results more intuitive, the optimal results under the same reference indicator are indicated in bold, as shown in Table 2.

By analyzing the experimental results in Table 2, it can be seen that, within a certain range, increasing the resolution of the input image is beneficial to improve the accuracy and recall of model detection. In other words, the input image resolution is positively correlated with the model performance. However, when increasing the input image resolution from 640 × 640 pixels to 768 × 768 pixels, not only did it hardly bring any improvement in model performance, but it also increased the inference time of the model. Considering the model performance and model speed, the model input image resolution of 640 × 640 pixels is more appropriate. Therefore, all subsequent experimental results were obtained at an input image resolution of 640 × 640 pixels. Overall, the impact of input image resolution on the model’s detection precision and recall is limited. Even under low-resolution conditions of 256 × 256 pixels, the model’s precision and recall still reach 0.919 and 0.841, respectively. This basically meets the requirements for weld-crack-defect detection in industrial scenarios.

In order to investigate the practical impact of input image illumination on the model detection performance, this paper systematically tested the model for detection precision and recall under three different exposure times: low, medium and high. The experimental results are shown in Table 3. The comprehensive evaluation results show that the illumination variations in the input images produce relatively small effects on the detection precision and recall of the model. This indicates that the model exhibits strong robustness to illumination variations and maintains good performance in variable welding operation environments. The model detection accuracy, recall and mAP@0.5 metrics were lowest under the low-illumination input image test conditions.

**Figure 5:**
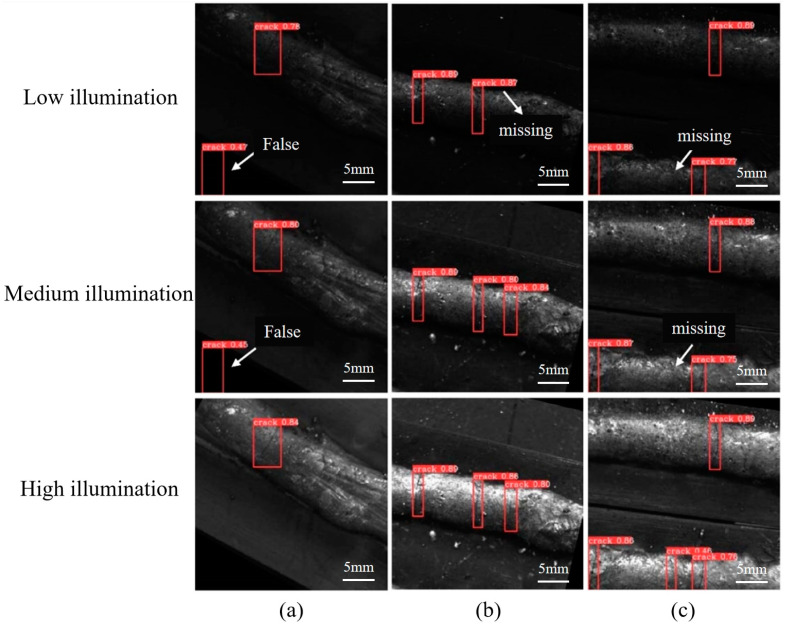
Detection results of weld-crack defects under different illumination levels. (**a**) Group One; (**b**) Group Two; (**c**) Group Three.

The test results of the model on the input images under different exposure times are shown in Figure 5. From the overall detection results, the weld-crack-defect detection model is more adaptable to illumination changes, and its average detection precision and recall show good stability. However, if focusing on the details of a single detection, it can be noticed that the model is more prone to missed and false detections when dealing with images in low- and medium-illumination conditions compared to high-illumination conditions.

The results of the performance comparison between the proposed weld-crack-defect detection model YOLOv6-NW and other advanced target detectors on the weld-crack-defect detection task are shown in Table 4. YOLOv6-NW significantly outperforms YOLOv5 both in model detection performance and model size. Compared with YOLOv6-N, the number of model parameters of YOLOv6-NW is only 16% of that of YOLOv6-N, but the model detection precision and recall still reach a comparable level with YOLOv6-N. Although YOLOv6-NW is 0.022 lower than YOLOv7-T in terms of model recall, its model parameter count is only 12% of YOLOv7-T. This means YOLOv6-NW shows higher competitiveness and practicality in industrial environments with limited arithmetic resources.

**Figure 6:**
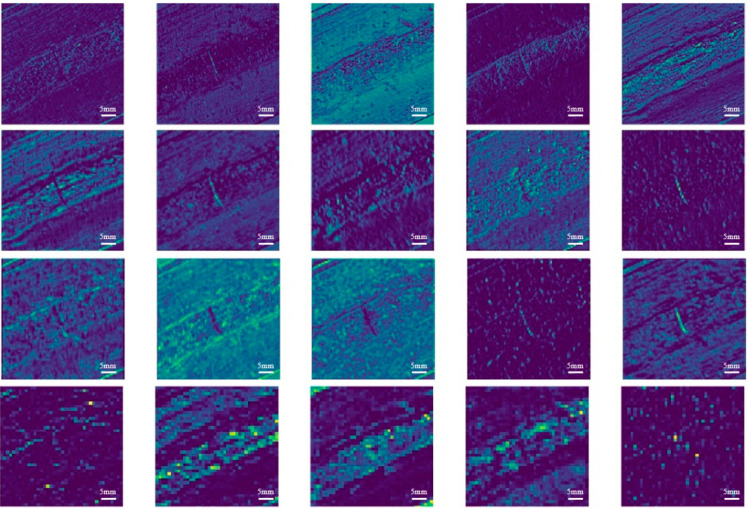
Feature-map visualization.

The results of the visualization of the output feature maps of some of the network layers of YOLOv6-NW are shown in Figure 6. From the first line, it can be seen that the feature maps output by the shallow network have significant visual differences, with obvious edges. The crack area of the weld seam has good separability from the normal and background areas of the weld seam. As the number of layers of the deep neural network gradually increases, the feature maps in the second row show a trend that the detail information begins to decrease. Although the crack region is still visible, its edges become slightly softer and no longer sharp as in the shallow feature maps. This indicates that the network starts to gradually filter out some non-critical details at this level. Entering the third row, the details in the feature maps further decrease, while the content of abstract semantic information increases significantly. At this time, the crack region of the weld is no longer only dependent on the edge or texture change to distinguish, but more depends on the abstract semantic features related to the crack. These features may not be visually intuitive, but they are crucial for the subsequent processing of the network. By the fourth line, the edges of the output feature map have become rather blurry and abstract. The feature maps at this level contain more high-level semantic feature information, which is crucial for understanding the overall content and structure of the image. The crack regions of the weld may no longer appear as distinct edge or texture changes at this level, but exist in a more abstract and global way with semantic features related to the crack.

## 5. Conclusions

This paper mainly focuses on the research of weld-crack detection technology based on target recognition. Firstly, an unnatural weld-crack preparation method is proposed. Secondly, data labelling methods and several data augmentation strategies are introduced. Finally, a lightweight weld-crack-defect detection model, YOLOv6-NW, was developed for industrial needs. The SIoU loss function with an angle perception penalty term is introduced to improve the model convergence speed and enhance the model detection performance. And, the mixed-illumination weld-crack images are used in the model training to enhance its robustness to the complex and variable arc-welding operation scenarios. The experimental results show that the model performance of YOLOv6-NW degrades to a lesser extent at low resolutions and still maintains good detection accuracy. In addition, the model is not sensitive to changes in illumination, indicating good robustness. The proposed weld-crack-defect detection model, YOLOv6-NW, not only has the advantage of being a lightweight model, but also has comparable performance to other state-of-the-art algorithms.

## Figures and Tables

**Table 1:** Structural differences between YOLOv6-N and YOLOv6-NW.

Unit Structure	YOLOv6-N	YOLOv6-NW
RepBlock #2	RepVGGBlock + 1 × RepVGGBlock	RepVGGBlock
RepBlock #3	RepVGGBlock + 3 × RepVGGBlock	RepVGGBlock + 1 × RepVGGBlock
RepBlock #4	RepVGGBlock + 5 × RepVGGBlock	RepVGGBlock + 2 × RepVGGBlock
RepBlock #5	RepVGGBlock + 1 × RepVGGBlock	RepVGGBlock
RepBlock #p4	RepVGGBlock + 3 × RepVGGBlock	RepVGGBlock + 1 × RepVGGBlock
RepBlock #p3	RepVGGBlock + 3 × RepVGGBlock	RepVGGBlock + 1 × RepVGGBlock
RepBlock #n3	RepVGGBlock + 3 × RepVGGBlock	RepVGGBlock + 1 × RepVGGBlock
RepBlock #n4	RepVGGBlock + 3 × RepVGGBlock	RepVGGBlock + 1 × RepVGGBlock

**Table 2:** Performance comparison at different input image resolutions.

Input Resolution (Pixels)	Precision (P)	Recall (R)	mAP@0.5
256 × 256	0.919	0.841	0.889
512 × 512	0.933	0.856	0.921
640 × 640	0.963	**0.884**	**0.949**
768 × 768	**0.964**	0.882	0.948

**Table 3:** Comparison of performance under different illumination images.

Exposure Time (ms)	Precision (P)	Recall (R)	mAP@0.5
Low (50)	0.952	0.875	0.936
Medium (150)	**0.963**	0.886	0.953
High (300)	0.961	**0.906**	**0.959**

**Table 4:** Performance comparison of YOLOv6-NW with other algorithms.

Network	Precision (P)	Recall (R)	mAP@0.5	Params
YOLOv5-N	0.926	0.859	0.923	1.7 M
YOLOv6-N	0.968	0.882	0.946	4.3 M
YOLOv7-T	0.953	**0.907**	0.942	5.9 M
YOLOv6-NW	**0.969**	0.885	**0.949**	**0.7 M**

## Data Availability

The code and data can be obtained by contacting the corresponding author through a reasonable request. Please note that these resources are not publicly accessible to ensure privacy.
